# Prevalence of Psychiatric Illness Among Keratoconus Patients

**DOI:** 10.7759/cureus.42141

**Published:** 2023-07-19

**Authors:** Fahad Alfardan, Meznah H Alsanad, Haifa A Altoub

**Affiliations:** 1 Department of Surgery, Division of Ophthalmology, National Guard Hospital, Riyadh, SAU; 2 College of Medicine, King Saud Bin Abdulaziz University for Health Sciences, Riyadh, SAU

**Keywords:** corneal transplant, anxiety, depression, psychiatry & mental health, keratoconus (kc)

## Abstract

Background

Keratoconus is a chronic corneal disorder characterized by progressive thinning of the cornea resulting in visual deterioration. Keratoconus patients have a higher rate of psychiatric morbidities. Therefore, this study will assess the prevalence of psychiatric illness among keratoconus patients.

Methods

We conducted a quantitative retrospective study in three centers across Saudi Arabia from June 2016 to February 2023. We included all patients previously diagnosed with keratoconus and any psychiatric illness. We excluded patients with other ocular diseases in addition to keratoconus. After we extracted the data, we coded and analyzed them using IBM SPSS Statistics for Windows, Version 21.0 (2012; IBM Corp., Armonk, New York, United States) and GraphPad Prism Version 8.4.2 (Dotmatics, Boston, Massachusetts, United States).

Results

The study included 57 keratoconus patients, with the majority being Saudi (96.5%), female (2:1), in the age group of 31-40 years (40.4%), and with a high-school level of education. The majority of patients were also obese (42.4%) and non-smokers (82%). Most patients had comorbid medical disorders. Of the 57 patients, 63.2% had anxiety disorders, 56.1% had depression, 10.5% had schizophrenia, and 1.8% had bipolar disorder. Patients with only a primary-school level of education were significantly more likely to be diagnosed with bipolar disorder, whereas those who were single were significantly more likely to be diagnosed with schizophrenia. Corneal transplant was also significantly associated with schizophrenia. Most patients (51%) were diagnosed with a psychiatric illness before keratoconus was diagnosed.

Conclusion

Among the keratoconus patients, single relationship status, primary-school level of education, and comorbidities were significantly associated with schizophrenia, bipolar disorder, and anxiety disorder, respectively. In addition, corneal transplant was significantly associated with schizophrenia. Lastly, the occurrence of psychiatric illness before keratoconus diagnosis was observed in the majority of patients.

## Introduction

Keratoconus is a chronic corneal disorder characterized by progressive thinning and steepening of the cornea resulting in visual deterioration and irregular astigmatism [1]. Although the disorder has a bilateral and asymmetric corneal involvement, usually one eye is more severely affected than the other [2]. Typically, keratoconus starts to develop in the second and third decades of life, progressing further until the fourth decade [1]. Its exact etiology and pathogenesis are still unknown; however, family history of keratoconus, eye-rubbing, and atopy are well-recognized risk factors [3]. A recent meta-analysis found that the global prevalence of keratoconus was 1.38 per 1000 people [3]. Keratoconus prevalence varies widely depending on ethnicity and geographical areas, being as low as 0.2 per 100,000 people in Russia and over 4790 per 100,000 people in Saudi Arabia [4,5].

Chronic diseases may negatively impact one’s psychosocial life, especially if they occur during the critical periods of adolescence and early adulthood, in which people develop physically, cognitively, and psychosocially [6]. Studies on personality features among patients with keratoconus have revealed different common traits, including paranoia, skepticism, and insecurity [5]. However, according to one review article, keratoconus patients have no unique personality structure [7]. Furthermore, keratoconus patients have a higher rate of psychiatric morbidities, such as obsessive-compulsive disorder, generalized anxiety disorder, and major depression, with the latter being the most frequently reported [6-8]. Although several studies have shown an association between keratoconus and depression, one cohort study rejected such an association [9,10]. In addition, there have been reports of cases of schizophrenia among keratoconus patients [11-13]. A local study has evaluated the prevalence of depression among keratoconus in Saudi Arabia, revealing that 40.6% of patients have depression [9]. In comparison, psychiatric hospital clinics in Saudi Arabia reported a total of 397,210 cases of mental illness in 2017, accounting for 1.22% of the entire population that year [14]. Few studies have assessed the prevalence of psychiatric illnesses in patients diagnosed with keratoconus. Therefore, this study aimed to assess the prevalence of psychiatric illness among keratoconus patients.

## Materials and methods

This quantitative retrospective multicenter study was conducted in King Abdulaziz Medical City (KAMC), Riyadh, KAMC, Jeddah, and Prince Mohammed bin Abdulaziz Hospital, Al-Madinah, Saudi Arabia. KAMC in Riyadh is considered one of the most comprehensive healthcare medical cities in Saudi Arabia, and its current workforce includes 2451 physicians, dentists, and residents. KAMC in Jeddah has a capacity of 751 beds and provides medical services to those living in the Western Region of Saudi Arabia. Prince Mohammed bin Abdulaziz Hospital in Al-Madinah, with a 215-bed capacity, is renowned for its high-quality care. King Abdullah International Medical Research Center approved the study (approval number: IRB/2774/22).

We included all patients diagnosed with both keratoconus and any psychiatric illness in the study. We excluded patients with other ocular diseases in addition to keratoconus from the study. We identified a total of 769 patients with keratoconus at KAMC in Riyadh, of which 51 met the inclusion criteria and were included in the study. Additionally, we identified 443 patients with keratoconus at KAMC in Jeddah, with only three meeting the inclusion criteria. Lastly, we identified 57 patients with keratoconus at Prince Mohammed bin Abdulaziz Hospital, Al-Madinah, of which only three met the inclusion criteria. 

As our study was a chart review, we used a non-randomized consecutive sampling technique, and we took all subjects for distributive purposes. We collected the data using chart review. We then reviewed and extracted the patients’ data from the BESTCare electronic medical record (EMR) system in an electronic form and entered them into a Microsoft Excel file (Microsoft Corporation, Redmond, Washington, United States). The variables included the patients’ demographics, date of keratoconus diagnosis, type of keratoconus treatment if any, date of psychiatric illness diagnosis, and type of psychiatric illness diagnosed based on the Diagnostic and Statistical Manual of Mental Disorders, Fifth Edition, Text Revision (DSM-5-TR). The psychiatric illnesses that were assessed are depression, bipolar disorder, schizophrenia, anxiety disorders (including obsessive-compulsive disorder, generalized anxiety disorder, and phobia), dysthymia, and panic attacks.

We used IBM SPSS Statistics for Windows, Version 21.0 (2012; IBM Corp., Armonk, New York, United States) and GraphPad Prism Version 8.4.2 (Dotmatics, Boston, Massachusetts, United States) to perform the statistical analysis. We performed the Kolmogorov-Smirnov test to check the normality of the variables. We expressed descriptive statistics as means, standard deviations, numbers, and percentages according to the type of data. We conducted chi-squared tests to assess the relationship between psychiatric illness and demographic and clinical characteristics of the keratoconus patients. We used independent t-tests for the numerical data, where a p-value of <0.05 was considered statistically significant.

## Results

Out of 1269 patients with keratoconus diagnosed between 2016 and 2023, we included only 57 keratoconus patients with psychiatric illness in this study (4.5% of the total keratoconus patients). There were more female than male patients (2:1). The largest age group was 31-40 years (40.4%). The mean BMI of the patients was 31.67, which falls within the obese category. The majority of the patients were Saudi (96.5%), married (49.1%), and non-smokers (82%). Most patients had a high-school level of education, and 48% were employed (Table 1).

**Table 1:** Demographic characteristics of the patients (n=57).

Variable	n (%)
Gender	
Male	20 (35.1%)
Female	37 (64.9%)
Age (years)	
18-30	19 (33.3%)
31-40	23 (40.4%)
>40	15 (26.3%)
BMI (mean ± SD)	31.67 ± 18.19
Nationality	
Saudi	55 (96.5%)
Non-Saudi	2 (3.5%)
Marital Status	
Single	20 (35.1%)
Married	28 (49.1%)
Divorced	8 (14%)
Widow	1 (1.8%)
Smoking Status	
Smoker	9 (18%)
Non-smoker	41 (82%)
Missing	7
Level of Education	
Primary School	4 (7.3%)
Middle School	6 (10.9%)
High School	18 (32.7%)
Bachelor’s Degree	16 (29.1%)
Diploma	5 (9.1%)
None	3 (5.5%)
Missing	3
Employment Status	
Employed	27 (48.2%)
Unemployed	24 (42.9%)
Student	2 (3.6%)
Missing	4

We calculated the mean visual acuities of the right and left eyes of the keratoconus patients, which showed no significant differences (Table 2). The presence of comorbid medical disorders along with keratoconus was 87.5%, with dyslipidemia being the most common comorbid disorder among these patients followed by hypothyroidism and bronchial asthma; 41.1% of patients were using corrective eyeglasses and 28.6% were using corrective contact lenses; 70.4% of patients underwent no surgery followed by 27.8% of patients who underwent corneal transplantation. Furthermore, the most common procedure used for surgery was crosslinking (21.8%) followed by intrastromal ring insertion and both comorbid procedures (i.e., crosslinking and intrastromal ring) (5.5% each) (Table 3).

**Table 2:** Visual acuities of patients with keratoconus. CDVA: corrected distance visual acuity; UVDA: uncorrected distance visual acuity; Km: mean keratometry; Kmax: maximum anterior sagittal curvature; K1: flat keratometry; K2: steep keratometry

Variable	Mean ± SD
Right eye	
CDVA	0.746 ± 0.21
UVDA	0.5138 ± 0.3511
K1	46.47 ± 7.669
K2	50.48 ± 9.738
Km	48.25 ± 8.213
Kmax (PW1)	55.50 ± 12.84
Thinnest pachymetry	470.3 ± 77.05
Left eye	
CDVA	0.6482 ± 0.243
UVDA	0.9516 ± 3.28
K1	47.92 ± 7.447
K2	51.38 ± 7.667
Km	49.53 ± 7.308
Kmax	58.92 ± 13.5
Thinnest pachymetry	448.6 ± 65.82

**Table 3:** Disorders, surgeries, and procedures used for keratoconus patients. ICL: implantable collamer lens

Variables	n (%)
Comorbid Medical Disorders	
Yes	42 (87.5%)
No	6 (12.5%)
Missing	9
Use of Corrective Eyeglasses	
Yes	23 (41.1%)
No	33 (58.9%)
Missing	1
Use of Corrective Contact Lenses	
Yes	16 (28.6%)
No	40 (71.4%)
Missing	1
Eye Surgery	
Corneal transplant	15 (27.8%)
Right eye	3 (20%)
Left eye	3 (20%)
Both eyes	1 (6.7%)
Other (ICL both eyes)	1 (1.9%)
No surgery	38 (70.4%)
Missing	3
Eye Procedure	
Crosslinking	12 (21.8%)
Intrastromal corneal ring	3 (5.5%)
No procedure	37 (67.3%)
Two combined procedures (crosslinking and intrastromal corneal ring)	3 (5.5%)
Missing	2

Among the different psychiatric illness diagnoses in these 57 patients, the majority (63.2%) had anxiety disorder, 56.1% had depression, and 10.5% had schizophrenia. Only 1.8% of patients had bipolar disorder, and 36.8% showed multiple comorbid psychiatric illnesses. Depression and anxiety disorder were the most common diagnoses among those with multiple comorbid psychiatric illnesses (Table 4).

**Table 4:** Prevalence of psychiatric illness in keratoconus patients.

Variable (n=57)	n (%)
Diagnosis of Depression	
Yes	32 (56.1%)
No	25 (43.9%)
Diagnosis of Bipolar Disorder	
Yes	1 (1.8%)
No	56 (98.2%)
Diagnosis of Schizophrenia	
Yes	6 (10.5%)
No	51 (89.5%)
Diagnosis of Anxiety Disorder	
Yes	36 (63.2%)
No	21 (36.8%)
Diagnosis of Other Disorders	
Yes	3 (5.3%)
No	54 (94.7%)
Diagnosis of Multiple Comorbid Psychiatric Illnesses	
Yes	21 (36.8%)
No	36 (63.2%)

According to our analysis, only two associations between demographic characteristics and psychiatric illness diagnoses were statistically significant. Being single had a statistically significant relationship with schizophrenia diagnosis, and patients with only a primary-school level of education had a statistically significant relationship with bipolar diagnosis (X2=8.62, p-value=0.034; X2=12.98, p-value=0.04, respectively). All other variables had no statistically significant association with psychiatric illness (Table 5).

**Table 5:** Association between demographic characteristics of keratoconus patients with psychiatric illness. For independent samples t-tests and chi-squared tests, *p<0.05 is considered statistically significant

Variable	Depression		X^2^	p-value	Bipolar		X^2^	p-value	Schizophrenia		X^2^	P-value	Anxiety disorder		X^2^	p-value	Other		X2	p-value
	Yes (32)	No (25)			Yes (1)	No (56)			Yes (6)	No (51)			Yes (36)	No (21)			Yes (3)	No (54)		
Gender																				
Male	12	8	0.19	0.67	0	20	0.55	0.46	1	19	0.99	0.31	14	6	0.62	0.43	1	19	0.004	0.94
Female	20	17			1	36			5	32			22	15			2	35		
Age (Years)																				
18–30	10	9	3.36	0.19	0	19	2.85	0.24	0	19	3.82	0.15	15	4	3.09	0.21	1	18	0.98	0.92
31–40	16	7			0	23			3	20			13	10			1	22		
>40	6	9			1	14			3	12			8	7			1	14		
BMI (mean ± SD)	28.61±9.4	35.6±25.0		0.15	1±31.7	56±31.6		0.99	6±32.9	51±31.5		0.71	36±32.2	21±30.7		0.76	3±29.4	53±31.8		0.82
Nationality																				
Saudi	31	24	0.03	0.86	1	54	0.04	0.847	5	50	3.43	0.64	35	20	0.15	0.69	3	52	0.11	0.73
Non-Saudi	1	1			0	2			1	1			1	1			0	2		
Marital status																				
Single	13	7	2.62	0.45	0	20	1.05	0.78	0	20	8.65	0.034*	14	6	1.65	0.65	1	19	0.70	0.87
Married	13	15			1	27			3	25			17	11			2	26		
Divorced	5	3			0	8			3	5			4	4			0	8		
Widow	1	0			0	1			0	1			0	1			0	1		
Smoking status																				
Smoker	6	4	0.081	0.78	0	10	0.25	0.61	1	9	0.04	0.82	5	5	1.42	0.23	0	10	0.52	0.47
Non-smoker	22	18			1	39			5	35			28	12			2	38		
Level of education																				
Primary school	2	2	6.99	0.32	1	3	12.98	0.04*	0	4	11.84	0.07	2	2	10.78	0.9	0	4	6.52	0.36
Middle school	3	3			0	6			3	3			3	3			0	6		
High school	7	11			0	18			1	17			12	6			3	15		
Bachelor’s degree	11	5			0	16			2	14			9	7			0	16		
Diploma	4	1			0	5			0	5			5	0			0	5		
None	2	1			0	3			0	3			3	0			0	3		
Employment status																				
Employed	15	12	2.41	0.49	0	27	1.35	0.71	4	23	1.22	0.75	17	10	5.85	0.12	3	24	3.40	0.33
Unemployed	13	11			1	23			2	22			17	7			0	24		
Student	1	1			0	2			0	2			1	1			0	2		

We analyzed the associations between eye disorders, surgery, procedure type, and psychiatric illness diagnoses by using the chi-squared test. The presence of comorbid disorders in keratoconus patients showed a significant relationship with diagnoses of anxiety disorder and other psychiatric illnesses (X2=11.7, p=0.001; X2=6.26, p=0.012, respectively). In addition, corneal transplants had a statistically significant relationship with schizophrenia (X2=8.31, p-value=0.01). All other variables had no statistically significant association with psychiatric illness (Table 6).

**Table 6:** Association between eye disorders and surgery procedure of keratoconus patients with psychiatric illness. For independent samples t-tests and chi-squared tests, *p <0.05 and **p <0.001 are considered statistically significant, respectively.

Variable	Depression		X^2^	p-value	Bipolar		X^2^	p-value	Schizophrenia		X^2^	p-value	Anxiety disorder		X^2^	p-value	Other		X2	p-value
	Yes (32)	No (25)			Yes (1)	No (56)			Yes (6)	No (51)			Yes (36)	No (21)			Yes (3)	No (54)		
Comorbid medical disorders																				
Yes	23	19	4.49	0.03*	1	42	0.14	0.70	5	38	0.77	0.37	31	12	11.77	0.001**	0	43	6.26	0.012*
No	6	0			0	6			0	6			0	6			1	6		
Use of corrective eyeglasses																				
Yes	12	11	0.16	0.68	0	23	0.71	0.4	2	21	0.16	0.68	15	8	0.12	0.72	2	21	0.85	0.35
No	19	14			1	32			4	29			20	13			32	1		
Use of corrective contact lenses																				
Yes	8	8	0.26	0.61	1	15	2.54	0.11	1	15	0.46	0.49	10	6	0.000	1.0	2	14	2.25	0.13
No	23	17			0	40			5	35			25	15			1	39		
Eye surgery																				
Corneal transplant	9	6	1.39	0.49	1	14	2.64	0.26	1	14	8.31	0.01*	6	9	5.96	0.051	2	13	2.40	0.3
Other (ICL both eyes)	0	1			0	1			1	0			0	1			0	1		
No surgery	22	16			0	38			4	34			27	11			1	37		
Eye procedure																				
Cross linking	9	3	2.70	0.25	0	12	0.41	0.81	1	11	0.65	0.72	6	6	0.62	0.73	1	11	0.33	0.84
Intrastromal corneal ring	1	2			0	3			0	3			2	1			0	3		
No procedure	19	18			1	36			5	32			23	14			2	35		

We used Student’s t-test to examine the correlation between previous diagnoses of psychiatric illness and that of keratoconus. Fifty-one percent of patients in this study were diagnosed with psychiatric illness before being diagnosed with keratoconus, whereas 49% were diagnosed after. Therefore, the majority of patients were diagnosed with psychiatric illness before keratoconus (p<0.001).

## Discussion

Our study demonstrated a statistically significant association between single patients with keratoconus and schizophrenia. Although a local study by Al Qahtani et al. did not find an association between sociodemographic characteristics and keratoconus, Bak-Nielson et al. reported that single individuals are at a higher risk of keratoconus [15,16]. This result might be explained by the social and cognitive impairment caused by schizophrenia, which is compounded by stigma and discrimination, leading to a higher likelihood of being single.

Although local studies reported a trend of secondary-school level of education or higher among keratoconus patients [15], our findings showed a significant association between primary-school level of education and bipolar disorder in keratoconus patients. This result could be attributed to the functional impairment caused by psychiatric illness, in addition to the reduced visual acuity associated with keratoconus, leading to difficulties in pursuing education.

Our study showed that 63.2% of the keratoconus patients had anxiety disorders, which was consistent with recent studies linking keratoconus and anxiety [6]. However, we also found that the presence of comorbid medical disorders in keratoconus patients was a significant factor in the development of anxiety disorder, affecting 87.5% of our patients. The association between chronic medical comorbidities and anxiety was also demonstrated in a study by Baghdadi et al. based in Saudi Arabia [17]. It is difficult due to the nature of the study to determine whether anxiety developed due to keratoconus alone or having other chronic medical comorbidities.

Our study found a statistically significant association between schizophrenia and having a corneal transplant, which is a treatment option offered for patients with advanced keratoconus [1]. This result might suggest that advanced keratoconus is associated with schizophrenia. Similarly, a case was reported in 2003 of a keratoconus patient diagnosed with schizophrenia who also had a corneal transplantation [13]. Many hypothesizes have been proposed to explain the co-incidence of advanced keratoconus and schizophrenia including having a similar underlying pathophysiology. For instance, interleukin-6 (IL-6), tumor necrosis factor-α (TNF-α), and oxidative stress have been found in high levels in both keratoconus and schizophrenia patients [12]. Furthermore, genetic components have also been proposed to be linked in both keratoconus and schizophrenia including variants at 13q32 and a locus mapped to chromosome 21 [12,13]. Lastly, the diagnosis of keratoconus might cause stress/anxiety which in turn may contribute to the development of psychotic disorders [12].

Studies have shown that keratoconus patients have a higher likelihood of developing psychiatric illness [6-9]. In one study, 37.2% of keratoconus patients were diagnosed with psychiatric illness, with depression being the most common followed by obsessive-compulsive disorder and social phobia [6]. Another local study has demonstrated that 40.6% of keratoconus patients have depression [9]. Nevertheless, our study was the first to assess the chronological relationship between keratoconus and psychiatric illness. We found that the diagnosis of psychiatric illness was made before the diagnosis of keratoconus in 51% of patients, with a statistically significant p-value. This result signifies the potential predisposition of keratoconus patients in having a psychiatric illness, rather than psychiatric illness being a result of the chronicity of the disease. Further studies are needed to assess the co-occurrence of these two diseases.

One of the limitations of our study was its small sample size, as it possibly hindered the detection of significant associations between variables. This limitation was largely because of the under-detection of psychiatric illness in Saudi Arabia due to the stigma towards psychiatric illness, which can make many patients hesitant to seek a psychiatric help [18]. The retrospective nature may limit the ability to establish causality between the diagnosis of keratoconus and psychiatric illness. Additionally, there may be other factors beyond those examined in this study that contribute to the development of psychiatric illness in keratoconus patients.

## Conclusions

Several key findings have been identified in this study. Among keratoconus patients, single relationship status, primary-school level of education, and comorbidities were significantly correlated with schizophrenia, bipolar, and anxiety disorders, respectively. In addition, corneal transplant and schizophrenia had a significant association. Lastly, the occurrence of psychiatric illness before keratoconus diagnosis was observed in the majority of patients. These findings emphasize the importance of psychiatric screening among keratoconus patients. Ophthalmologists should consider asking patients about their mental and emotional wellbeing, as this action could help to identify those requiring psychiatric referral.
